# Predictors of mental health and academic outcomes in first-year university students: Identifying prevention and early-intervention targets

**DOI:** 10.1192/bjo.2020.24

**Published:** 2020-05-08

**Authors:** A. Duffy, C. Keown-Stoneman, S. Goodday, J. Horrocks, M. Lowe, N. King, W. Pickett, S. H. McNevin, S. Cunningham, D. Rivera, L. Bisdounis, C. R. Bowie, K. Harkness, K. E. A. Saunders

**Affiliations:** Department of Psychiatry, Division of Student Mental Health, Queen's University, Kingston, Canada; and Department of Psychiatry, University of Oxford, UK; Dalla Lana School of Public Health, University of Toronto, Canada; Department of Psychiatry, University of Oxford, UK; Department of Mathematics and Statistics, University of Guelph, Canada; Department of Mathematics and Statistics, University of Guelph, Canada; Department of Public Health Sciences, Queen's University, Canada; Department of Public Health Sciences, Queen's University, Canada; Department of Psychiatry, Division of Student Mental Health, Queen's University, Canada; Department of Psychology, Queen's University, Canada; Department of Psychiatry, Division of Student Mental Health, Queen's University, Canada; Department of Psychiatry, University of Oxford, UK; Department of Psychology, Queen's University, Canada; Department of Psychology, Queen's University, Canada; Department of Psychiatry, University of Oxford, UK

**Keywords:** Student mental health, risk factors, anxiety symptoms, depressive symptoms, prevention and early-intervention

## Abstract

**Background:**

Although there is growing interest in mental health problems in university students there is limited understanding of the scope of need and determinants to inform intervention efforts.

**Aims:**

To longitudinally examine the extent and persistence of mental health symptoms and the importance of psychosocial and lifestyle factors for student mental health and academic outcomes.

**Method:**

Undergraduates at a Canadian university were invited to complete electronic surveys at entry and completion of their first year. The baseline survey measured important distal and proximal risk factors and the follow-up assessed mental health and well-being. Surveys were linked to academic grades. Multivariable models of risk factors and mental health and academic outcomes were fit and adjusted for confounders.

**Results:**

In 1530 students surveyed at entry to university 28% and 33% screened positive for clinically significant depressive and anxiety symptoms respectively, which increased to 36% and 39% at the completion of first year. Over the academic year, 14% of students reported suicidal thoughts and 1.6% suicide attempts. Moreover, there was persistence and overlap in these mental health outcomes. Modifiable psychosocial and lifestyle factors at entry were associated with positive screens for mental health outcomes at completion of first year, while anxiety and depressive symptoms were associated with lower grades and university well-being.

**Conclusions:**

Clinically significant mental health symptoms are common and persistent among first-year university students and have a negative impact on academic performance and well-being. A comprehensive mental health strategy that includes a whole university approach to prevention and targeted early-intervention measures and associated research is justified.

## Background

The transition to university coincides with a critical developmental period during which young people are expected to separate from family and take on increased responsibility for regulating their sleep, daily schedules and lifestyle; all while the brain is undergoing accelerated growth and shows increased sensitivity to stress.^1^ University students are exposed to numerous stressors related to financing their studies, making new friends and living away from home and local supports.^2^ At the same time, emergent adulthood represents the peak period of risk for onset of mental disorders^3^ associated with high morbidity, including university failure and drop-out,^4^ and increased mortality.^5^ Yet, successful completion of higher education is an important determinant of both healthy individual and societal growth and development.^6^ Taken together, entry to university represents a high-risk period for the development of significant mental health problems, but at the same time offers an important opportunity for effective prevention.^7^

Globally, university student populations are changing and more closely reflect the general population in terms of risk factors and rates of psychopathology, in part driven by increasing enrolment and widening access.^8,9^ For example, the World Health Organization Mental Health Survey reported comparable rates of common mental disorders in university student and non-student populations aged 18–22 years.^10^ Further, a recent study reported an increase in university student suicide rates since 2009 in England and Wales, although below that of the general non-student population of a similar age.^11^ Recent reports from varied higher education institutions and associations in both Canada and the UK have highlighted a substantial disparity between the increasing need for university student mental health support and available resources.^8,9,12^ Yet, there are little reliable data to inform the extent of mental health need in the transition to and over the course of university study or to identify salient targets for the development of evidence-informed preventive and early-intervention strategies.

## Aims

Research to date has been limited by a lack of longitudinal studies, low response rates yielding non-representative samples, and the use of varied and not always validated measures.^7,12^ Therefore, the primary aims of this longitudinal study were to (a) estimate the extent and persistence of clinically significant mental health symptoms; and (b) examine the importance of candidate potentially modifiable psychosocial and lifestyle risk factors for mental health and university-related outcomes in a large representative cohort of undergraduate students entering a major Canadian university.

## Method

### Overview of study design

The authors assert that all procedures contributing to this work comply with the ethical standards of the relevant national and institutional committees on human experimentation and with the Helsinki Declaration of 1975, as revised in 2008 and was approved by the Queen's University and Affiliated Teaching Hospitals Research Ethics Board (HSREB PSIY-609-18).

Details of the study protocol are published elsewhere.^13^ Briefly, U-Flourish is a longitudinal prospective study of a large cohort of undergraduate students representative of the first-year student body at Queen's University.^14^ During the first few weeks of starting university (September 2018) all first-year students were sent a link via their student email to a Letter of Information and after providing consent completed the time 1 survey. This survey asked about demographic, family and personal health information and included brief validated measures of candidate distal and proximal risk factors including early loss, childhood adversity, social, psychological and lifestyle risk factors, as well as anxiety and depressive symptoms, suicidal ideation and lifetime suicide attempts and self-harm.

At the end of the academic year and before final exams (March 2019), students who completed the time 1 survey were sent a link to complete the time 2 survey that asked about anxiety and depressive symptoms, self-harm, suicidal thoughts and attempts, and university well-being. After final grades were posted, survey data were linked to grades abstracted from the university database.

### Measures

#### Proximal psychosocial and lifestyle risk factors at time 1

Proximal psychosocial and lifestyle risk factors at time 1 were measured by brief validated scales including: the Brief Perceived Stress scale,^15^ the Rosenberg Self-Esteem scale,^16^ the Social Support subscale of the Resiliency Scale for Adolescence^17^ and the Sleep Condition Indicator.^18^ The amount of exercise was reported as an ordinal variable (never, less than weekly, once a week, 2–3 times a week and ≥4 times a week). Substance misuse was defined as engaging in any of the following at least once a week over the past month: binge drinking (≥4 drinks on one occasion), cannabis use, use of non-prescribed sleeping or wake-up pills or stimulants, pain killers, opiates or other. Depressive and anxiety symptoms at time 1 were measured by the 9-item Patient Health Questionnaire (PHQ-9)^19^ and 7-item Generalized Anxiety Disorder assessment (GAD-7),^20^ respectively.

#### Primary mental health outcomes at time 2

Primary mental health outcomes at time 2 were defined as positive screens for clinically significant depressive and anxiety symptoms measured by the PHQ-9 and GAD-7 examined as binary outcomes using the established cut-off score of 10. Self-harm, suicidal ideation and attempts were measured as binary outcomes (yes/no) to questions from the Columbia Suicide Severity Rating Scale:^21^ ‘have you ever harmed yourself without the intent of ending your life’, ‘have you had any thoughts of ending your life’ and ‘have you made a suicide attempt.’ Owing to the bias associated with dichotomising variables and decreased power, we examined secondary mental health outcomes as total symptom scores on the PHQ-9 and GAD-7. Functional impairment related to depressive and anxiety symptoms was assessed by asking ‘how difficult have these symptoms made it for you to do your work, take care of things at home, or get along with others?’ rated from 0 (not difficult at all) to 3 (extremely difficult). Respondents rating 2 or 3 were considered to have significant impairment associated with their symptoms.

### Academic outcomes

Academic outcomes included cumulative grade point average (GPA) abstracted from the university database and well-being at university indexed by the School Connectedness subscale of the College Student Subjective Well-Being questionnaire^22^ at time 2.

### Statistical analysis

Chi-square tests were used to compare the time 1 participants (*n* = 3029) with the final analysis participants with complete data at both time 1 and time 2 (*n* = 1530). Linear mixed-effects models with interaction were used to assess differences between males and females in changes in continuous mental health outcomes over time in the analysis sample (*n* = 1530). Distributions of continuous outcomes were checked for normality. PHQ-9 and GAD-7 total scores were positively skewed and were normalised with a square root transformation, whereas cumulative GPA was negatively skewed and was normalised with a square root transformation. School connectedness scores were not skewed and did not require normalisation. All adjusted models had a variance inflation factor <4 suggesting multicollinearity was not a significant factor in the models.

Adjusted and unadjusted logistic regression models for binary mental health outcomes (PHQ-9 /GAD-7 clinical cut-offs and suicidal thoughts/attempts) and linear regression models for continuous outcomes (PHQ-9/GAD-7 total scores, cumulative GPA, School Connectedness score) were fit to determine associations between time 1 predictors and time 2 outcomes. For binary mental health outcomes, both new onset (positive screen at time 2 only) and persistent (positive screen at both time 1 and time 2) were included as positive outcomes in the primary analyses. As sensitivity analyses, additional models were fit investigating only novel and persistent mental health outcomes.

Multivariable models of mental health outcomes were adjusted for potential confounders that were not the focus of this analysis including age, gender, family and lifetime history of mental illness, childhood abuse and bullying. Multivariable models of academic outcomes were adjusted for age, gender and childhood abuse. Interactions for age and gender were analysed for each adjusted model by including interaction terms with each predictor. It was decided *a priori* not to adjust models for outcomes measured at time 2 with baseline measurements of the outcome at time 1. All statistics were completed using R version 3.6.1 64bit for Windows.^23^

## Results

### Descriptive analysis

#### Participants

A total of 3029 first-year undergraduate students (58%) completed the time 1 survey at entry to university and as reported elsewhere were representative of the 5242 eligible first-year undergraduate population.^14^ The current analysis included 1530 participants who completed both the time 1 and time 2 surveys and self-identified as either male or female (supplementary Fig. 1 available at https://doi.org/10.1192/bjo.2020.24). There was insufficient power to include other gender categories (total *n* = 19). In addition, 36 medical students were removed from the analysis of GPA as they received pass/fail grades. The final analysis sample (*n* = 1530) was comparable with the time 1 participants (*n* = 3029) in terms of age, lifetime history of mental disorder and exposure to early adversity (supplementary Table 1); however, more females, those with a family history of mental disorder, and those with less parental education were more likely to continue throughout the study.

#### Proximal psychosocial and lifestyle risk factors at entry to university (time 1)

Approximately one-fifth of students met screening thresholds for sleep problems (21%), with more females compared with males (23% *v.* 13%; *P* < 0.001) (supplementary Table 2). Close to half the students surveyed (48%) endorsed substance misuse, largely attributable to binge drinking; higher in males compared with females (52% *v.* 42%; *P* = 0.001). Further 60% of students exercised once per week or less, of which 16% indicated they ‘never’ exercised; males exercised more frequently than females (*P* < 0.001). Female students indicated higher levels of stress (*P* < 0.001) and social support (*P* = 0.02). The majority of students indicated what is considered normative levels of self-esteem, but 18% reported low self-esteem; especially females compared with males (20% *v.* 10%; *P* < 0.001). All proximal psychosocial and lifestyle risk factors, with the exception of substance misuse, were significantly associated with anxiety and depressive symptoms at time 1 (supplementary Table 3).

#### Clinically significant symptoms at entry (time 1) and completion of first year (time 2)

At entry to university, almost one-third of students screened positive for depressive (28%) and anxiety (33%) symptoms, which were associated with significant impairment in 45% and 47% of students, respectively (Table 1). At the end of the first year, the proportion of students with clinically significant depressive and anxiety symptoms increased to 36% and 39%, respectively (Table 1), with rates of associated impairment at 54% and 48%, respectively. Although females had a higher rate of positive screens for depressive and anxiety symptoms at both time points compared with males, the rate of increase in symptoms from time 1 to time 2 was comparable (supplementary Fig. 2).

**Table 1:** Mental health and academic outcomes at time 1 and time 2 by gender^a^

Outcomes	Time 1	Time 2		
	All (*n* = 3029)	All (*n* = 1530)	Female (*n* = 1125)	Male (*n* = 405)
Depressive symptoms, *n* (%)				
Minimal/none (0–4)	1156 (42.4)	500 (32.7)	332 (29.5)	168 (41.5)
Mild (5–9)	819 (30.0)	474 (31.0)	419 (27.2)	142 (35.1)
Moderate (10–14)	418 (15.3)	265 (17.3)	203 (18.0)	65 (15.3)
Moderately severe (15–19)	207 (7.6)	176 (11.5)	135 (12.0)	41 (10.1)
Severe (20–27)	126 (4.6)	115 (7.5)	100 (8.9)	15 (3.7)
Clinically significant depressive symptoms (PHQ-9 > 10)	751 (27.5)	556 (36.3)	438 (38.9)	118 (29.1)
PHQ-9 functionally impaired (2 or 3)	338 (45.1)	302 (54.3)	251 (57.3)	51 (43.2)
Anxiety symptoms, *n* (%)				
Minimal/none (0–4)	1034 (37.8)	487 (31.8)	309 (27.5)	178 (44.0)
Mild (5–9)	815 (29.8)	452 (29.5)	336 (29.9)	116 (28.6)
Moderate (10–14)	506 (18.5)	311 (20.3)	243 (21.6)	68 (16.8)
Severe (15–21)	383 (14.0)	280 (18.3)	237 (21.1)	43 (2.5)
Clinically significant anxiety symptoms (GAD-7 > 10)	889 (32.5)	591 (38.6)	480 (42.7)	111 (27.4)
GADS-7 functionally impaired (2 or 3)	414 (46.8)	281 (47.5)	234 (48.8)	47 (42.3)
Suicide-related thoughts and behaviours,^b^ *n* (%)				
Wished you were dead	929 (34.1)	333 (21.8)	258 (22.9)	75 (18.5)
Suicide-related thoughts	792 (29.0)	221 (14.4)	169 (15.0)	52 (12.8)
Suicide attempt	166 (6.1)	24 (1.6)	18 (1.6)	6 (1.5)
Self-harmed without intent to end life	479 (17.6)	91 (5.9)	75 (6.7)	16 (4.0)
Cumulative GPA (letter equivalent), *n* (%)				
0 (F)	–	1 (0.1)	1 (0.1)	0 (0)
0.66–0.99 (D−)	–	7 (0.5)	5 (0.5)	2 (0.5)
1–1.99 (D/D+/C−)	–	69 (4.6)	45 (4.1)	23 (6.0)
2–2.99 (C/C+/B−)	–	420 (28.1)	331 (30.0)	88 (23.0)
3–3.99 (B/B+/A−)	–	818 (54.7)	609 (55.1)	204 (53.3)
4+ (A/A+)	–	181 (12.1)	114 (10.3)	66 (6.0)
Student Well-Being^c^ (maximum score 112), mean (s.d.)	–	71.0 (15.7)	71.2 (15.3)	70.2 (16.8)

GAD-7, Generalized Anxiety Disorder assessment; PHQ-9, Patient Health Questionnaire.

a. Percentages are based on non-missing responses.

b. Time 1 represents lifetime prior to time 1, time 2 represents over the academic year.

c. Total score of the 16-item College Well-Being Questionnaire.

At entry to university 29% of students endorsed lifetime suicidal thoughts, 6% suicide attempts and 18% self-harm.^14^ While at university, 14% of students indicated having had suicidal thoughts, 1.6% having made a suicide attempt and 6% reported having self-harmed. Females had higher rates of suicidal thoughts and self-harm at time 2 compared to males, but unlike at time 1 these differences did not reach statistical significance (15% *v.* 13%, *P* = 0.323; 7% *v.* 4%, *P* = 0.063, respectively).

**Fig. 1:**
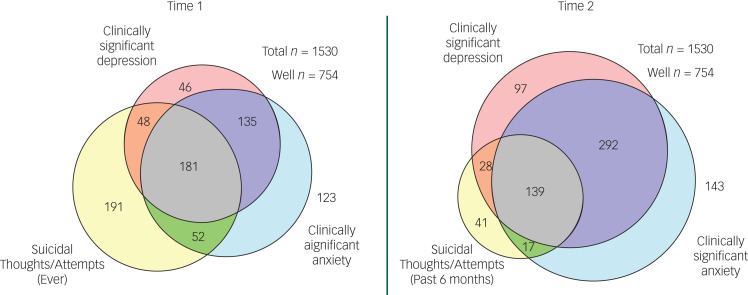
Overlap in mental health outcomes at entry to university (time 1) and at the end of the first year (time 2).

There was substantial overlap in mental health outcomes at both entry and completion of first year (Fig. 1). For example, 17% of students screened positive for both anxiety and depressive symptoms at entry and 23% had clinically significant anxiety and depressive symptoms and suicidal ideation/attempts. Similarly, at the end of first year 39% had clinically significant anxiety and depressive symptoms, and 18% screened positive for all three mental health outcomes.

The distribution of persistent mental health outcomes, present at both entry (time 1) and completion (time 2) of first year, compared with new-onset mental health outcomes, only present at time 2, was assessed (supplementary Table 4). Of the 556 students screening positive for depressive symptoms at time 2, 51% were persistent and 49% were new onset. Similarly, of 591 students screening positive for anxiety symptoms at time 2, 56% were persistent; and of the 225 students screening positive for suicidal ideation/attempts 79% were persistent. Furthermore, 27% of students with a negative screen for anxiety, depression or suicidal thoughts or attempts at entry to university, screened positive at time 2, whereas 29% of students who screened positive at time 1 no longer reported clinically significant mental health symptoms at time 2 (supplementary Fig. 3).

### Models of mental health outcomes at time 2

#### Associations between proximal risk factors at entry (time 1) and clinically significant symptoms at completion of first year (time 2)

All psychosocial and lifestyle risk factors at entry to university with the exception of substance use were significantly associated with a positive depressive screen at completion of first year (Table 2). Specifically, lower self-esteem, less frequent exercise, poorer sleep quality, higher perceived stress and less social support were associated with an increased risk of clinically significant depressive symptoms. These associations did not change after partial adjustments for gender, age and distal risk factors; while in the fully adjusted model taking into account all other risk factors in the model, there was no longer statistical significance for exercise frequency or social support.

**Table 2:** Unadjusted and adjusted associations between psychosocial and lifestyle factors (time 1) and positive screen for mental health (time 2; *n* = 1530) at the end of first-year university

Mental health outcomes (time 2), predictor (time 1)	Unadjusted		Partial adjustment^a^		Fully adjusted^b^	
	OR (95% CI)	*P*^c^	OR (95% CI)	*P*^c^	OR (95% CI)	*P*^c^
Positive depressive symptom screen (PHQ-9 >10)						
Self-esteem^d^	0.83 (0.81–0.85)	<0.001	0.85 (0.82–0.87)	<0.001	0.90 (0.87–0.93)	<0.001
Substance use	1.25 (1.02–1.54)	0.04	1.24 (0.99–1.54)	0.06	1.31 (1.02–1.69)	0.03
Exercise^d^	0.85 (0.79–0.93)	<0.001	0.87 (0.80–0.95)	<0.01	0.97 (0.88–1.06)	0.48
Sleep quality^d,e^	0.88 (0.87–0.90)	<0.001	0.89 (0.88–0.91)	<0.001	0.93 (0.91–0.95)	<0.001
Stress^d^	1.39 (1.33–1.46)	<0.001	1.34 (1.28–1.41)	<0.001	1.12 (1.05–1.19)	<0.01
Social support^d^	0.92 (0.90–0.95)	<0.001	0.92 (0.90–0.95)	<0.001	0.99 (0.96–1.03)	0.61
Positive anxiety symptom screen (GAD-7 >10)						
Self-esteem^d^	0.85 (0.83–0.87)	<0.001	0.87 (0.85–0.89)	<0.001	0.94 (0.91–0.97)	<0.001
Substance use	1.06 (0.86–1.30)	0.59	1.04 (0.83–1.29)	0.76	1.00 (0.79–1.28)	0.99
Exercise^d^	0.90 (0.83–0.97)	<0.01	0.92 (0.85–1.01)	0.07	1.04 (0.94–1.14)	0.45
Sleep quality^d,e^	0.89 (0.87–0.90)	<0.001	0.90 (0.88–0.92)	<0.001	0.94 (0.92–0.96)	<0.001
Stress^d^	1.42 (1.36–1.49)	<0.001	1.36 (1.30–1.44)	<0.001	1.21 (1.14–1.29)	<0.001
Social support^d^	0.94 (0.92–0.97)	<0.001	0.94 (0.92–0.97)	<0.001	1.01 (0.98–1.04)	0.69
Suicidal thoughts or attempts (yes)						
Self-esteem^d^	0.80 (0.77–0.83)	<0.001	0.82 (0.79–0.85)	<0.001	0.85 (0.81–0.89)	<0.001
Substance use	1.26 (0.95–1.68)	0.11	1.14 (0.84–1.54)	0.40	1.32 (0.95–1.83)	0.11
Exercise^d^	0.80 (0.71–0.89)	<0.001	0.81 (0.72–0.91)	<0.001	0.91 (0.80–1.04)	0.16
Sleep quality^d^^,^^e^	0.91 (0.89–0.93)	<0.001	0.93 (0.91–0.95)	<0.001	0.98 (0.95–1.01)	0.12
Stress^d^	1.40 (1.32–1.49)	<0.001	1.34 (1.26–1.43)	<0.001	1.09 (1.01–1.18)	0.04
Social support^d^	0.92 (0.89–0.95)	<0.001	0.92 (0.89–0.96)	<0.001	0.99 (0.95–1.04)	0.62

GAD-7, Generalized Anxiety Disorder assessment; PHQ-9, Patient Health Questionnaire.

a. Adjusted for: age, gender, physical and sexual abuse, bullied, family history for mental illness, and personal history of mental illness.

b. Adjusted for: age, gender, physical and sexual abuse, bullied, family history for mental illness, personal history of mental illness and all other predictors listed in the table.

c. *P*-value for test of association between outcome and predictor from logistic regression.

d. Continuous variable.

e. Sleep condition indicator.

Similarly, all candidate psychosocial and lifestyle risk factors at entry with the exception of substance use were associated with a positive anxiety screen at completion of first year (Table 2). After partial adjustments, exercise was no longer significant; whereas in the fully adjusted model substance use, exercise and social support were no longer statistically significant with clinically significant anxiety symptoms. Finally, all psychosocial and lifestyle risk factors at entry to university with the exception of substance use were associated with suicidal thoughts and/or attempts at completion of the first year (Table 2). Although there was no change after partial adjustments, in the fully adjusted model there was no longer evidence of an association between substance use, exercise, sleep and social support with suicidal ideation and/or attempts.

### Models of university outcomes at time 2

In unadjusted models, higher levels of depressive symptoms, suicidal thoughts or attempts, lower self-esteem and poorer sleep quality at entry to university predicted lower cumulative grades over the academic year (Table 3). Interestingly, in adjusted models, depressive symptoms remained associated with decreases in cumulative GPA; whereas anxiety symptoms appeared associated with increases in cumulative GPA. There was strong evidence that all predictors at time 1 were associated with school connectedness measured at time 2. In adjusted models, there remained evidence that depressive and anxiety symptoms, poorer self-esteem, higher perceived stress and lower social support were associated with lower levels of school connectedness.

**Table 3:** Unadjusted and adjusted association between proximal risk factors (time 1) and academic outcomes (time 2)

Academic outcomes (time 2) and predictor (time 1)	Unadjusted		Fully adjusted^a^	
	beta (95% CI)	*P*^b^	beta (95% CI)	*P*^b^
Cumulative GPA^c^^,^^d^ *n* = 1494				
Depressive symptoms^d^	−0.10 (−0.13 to −0.06)	<0.001	−0.14 (−0.20 to −0.07)	<0.001
Anxiety symptoms^d^	−0.04 (−0.07 to 0.002)	0.08	0.09 (0.03 to 0.15)	<0.01
Self-esteem^d^	0.06 (0.02 to 0.09)	<0.01	0.001 (−0.06 to 0.06)	0.98
Suicide-related thoughts	−0.93 (−1.38 to −0.48)	<0.001	−0.48 (−0.98 to 0.01)	0.05
Sleep quality^d,e^	0.07 (0.03 to 0.10)	<0.001	0.02 (−0.02 to 0.06)	0.39
Stress^d^	−0.08 (−0.16 to −0.003)	0.04	0.06 (−0.06 to 0.17)	0.34
Social support^d^	0.04 (−0.02 to 0.09)	0.17	0.002 (−0.05 to 0.06)	0.94
School connectedness^d^ (*n* = 1525)				
Depressive symptoms^d^	−0.96 (−1.09 to −0.83)	<0.001	−0.72 (−0.94 to −0.50)	<0.001
Anxiety symptoms^d^	−0.63 (−0.76 to −0.49)	<0.001	0.41 (0.21 to 0.62)	<0.001
Self-esteem^d^	1.05 (0.95 to 1.19)	<0.001	0.57 (0.37 to 0.77)	<0.001
Suicide-related thoughts	−7.08 (−8.75 to −5.41)	<0.001	−1.56 (−3.29 to 0.17)	0.08
Sleep quality^d^^,^^e^	0.55 (0.43 to 0.66)	<0.001	0.01 (−0.12 to 0.15)	0.84
Stress^d^	−1.76 (−2.04 to −1.48)	<0.001	−0.43 (−0.83 to −0.03)	0.03
Social support^d^	1.00 (0.80 to 1.20)	<0.001	0.44 (0.24 to 0.64)	<0.001

GPA, grade point average.

a. Adjusted for: age, gender, physical and sexual abuse and all other predictors in model.

b. *P*-value for test of association between outcomes and predictor for linear regression.

c. Outcome was normalised with a square transformation.

d. Continuous variable.

e. Sleep condition indicator.

### Secondary analyses

Whereas the main analysis focused on the association between potentially modifiable risk factors at entry to university and mental health and academic outcomes at the end of the first year, we explored and found evidence of an association between candidate distal risk factors (family and personal history of mental disorder, childhood abuse, neglect and peer bullying) and primary mental health outcomes at entry to university (supplementary Table 5).

Adjusted and unadjusted models were fit for new-onset positive screens for clinically significant depressive and/or anxiety symptoms and/or suicidal thoughts/attempts at time 2 (supplementary Table 6a). Additionally, adjusted and unadjusted models were fit for persistent positive screens for depressive and/or anxiety symptoms and/or suicidal thoughts/attempts present both at time 1 and at time 2 (supplementary Table 6b). Very little changed in this stratified analysis; however, there was insufficient evidence that any of the predictors were associated for new-onset suicidal thoughts/attempts after adjustments. Anxiety and depressive symptom total scores were explored with similar results (supplementary Table 7).

## Discussion

### Main findings

Approximately one-third of first-year undergraduate students endorsed clinically significant depressive and anxiety symptoms at entry to university and 36% and 39% respectively, met screening thresholds at the end of the academic year. At both time points, females had higher rates of clinically significant anxiety and depressive symptoms compared with males and almost a half of the students with positive symptom screens indicated a moderate-to-severe level of associated functional impairment. Further, 14% of students endorsed suicidal thoughts, 6% engaged in self-harm and 1.6% indicated at least one suicide attempt over the course of the academic year. Over 50% of students with clinically significant anxiety and depressive symptoms at entry showed persistence, meeting screening cut-offs again at the end of the academic year. The rate of persistence for suicidal ideation and/or attempts was even higher. There was a high degree of overlap across mental health outcomes at both time points.

**Fig. 2:**
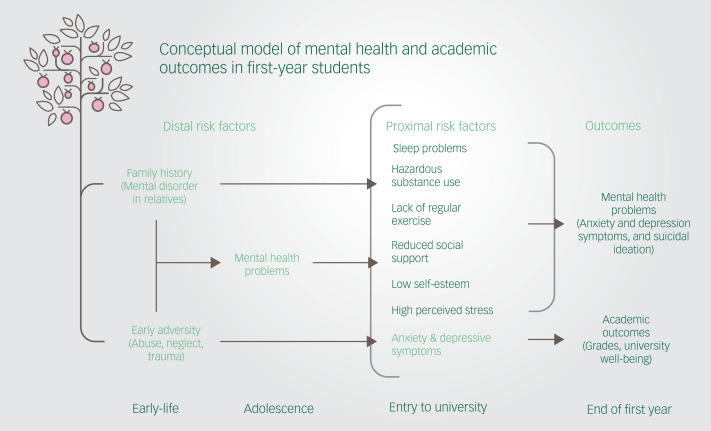
Conceptual model of the association between distal and proximal risk factors and mental health and academic outcomes.

Self-esteem, perceived stress, social support, sleep quality and exercise frequency at entry to university were associated with screening positive for mental health outcomes measured at the end of the first year. This finding held after adjusting for gender, age and important distal risk factors including lifetime and family history of mental disorders and childhood adversity (Fig. 2). Further, anxiety and depressive symptoms at the start of university were associated with lower grades over the year, whereas anxiety and depressive symptoms along with a number of psychosocial and lifestyle risk factors at entry to university were associated with lower levels of school connectedness at the end of the academic year. Taken together, findings underscore the extent to which symptoms at entry to university may have a negative impact on social and academic endeavours, which may serve to maintain clinically significant symptoms. Given the ubiquitous nature of mental health symptoms in the student population, mental health strategies should include whole university approaches and prioritise investment in rigorous prevention and early-intervention research to improve student mental health and well-being.

### Findings in context

Comparable rates of mental health outcomes have been reported in large cross-sectional studies of university students. For example, the World Health Organization Mental Health Survey study using structured interviews estimated that 20% of college students aged 18–22 met criteria for a 12-month mental disorder, with higher rates in high-income countries.^10^ Similarly, the World Mental Health International College Student Initiative reported that 35% of over 14 000 first-year students across eight countries screened positive for at least one lifetime mental disorder and 31% for at least one 12-month disorder.^24^ The Canadian data from the National College Health Assessment (NCHA) II survey reported 12-month rates of diagnosed anxiety and depressive disorders at 18% and 15%, respectively.^25^

Differences in rates of mental health outcomes in part reflect differences in methods and outcome variables. In this study, we used validated self-report measures and screening cut-off scores to estimate clinically significant symptoms, rather than structured interviews or self-reported diagnoses. In addition, participants in this study were largely adolescents who, compared with adults, have been associated with higher rates of positive screens for anxiety and depressive symptoms.^26,27^ Rates of suicidal thoughts and behaviour in our study are broadly comparable with the pooled 12-month prevalence estimates reported in a systematic review of 36 colleges worldwide^28^ and with Canadian NCHA data.^25^ A recent study of Spanish university students reported comparable 12-month prevalence and persistence of suicidal ideation; with a mood disorder diagnosis increasing risk and a higher sense of university membership having a protective effect.^29^ Peaks in student suicides have been associated with exam periods and winter months,^11^ which is relevant to our study as outcome data were collected just prior to final exams in March.

It is well established that university students commonly experience high levels of stress across financial, academic and social domains.^2^ Furthermore, student stress may be compounded by poor coping strategies and lifestyle choices such as binge drinking and low participation in physical exercise, recreational interest and hobbies.^1^ Although many symptomatic students do not develop severe mental illness, high stress is associated with sleep disturbance and distressing depressive and anxiety symptoms, as well as suicidal thoughts and behaviour; all of which have a negative impact on well-being and academic performance.^2,30^ Transition to university comes at a time of disrupted social networks,^1^ although a stronger sense of university connectedness appears protective for well-being, life satisfaction and academic performance outcomes.^31^ Moreover, university represents an important developmental stage characterised by separation and individuation, and for some the transcendence of earlier adversity, which can manifest as clinically significant symptoms.^32^

Interventions targeting stress, self-esteem, lifestyle factors and social support in clinical and general population samples have proven beneficial, yet there is limited research in university student populations. However, emerging evidence supports that healthy coping and stress resilience interventions using cognitive–behavioural therapy, behavioural and mindfulness approaches can be effective in university students, at least in the short term.^33–35^ The British Active Student survey reported that regular physical activity was associated with improved well-being, social inclusion and academic attainment.^36^ Further, systematic reviews of digital and face-to-face interventions in university students support small-to-moderate effect sizes on a wide variety of mental health symptoms and academic functioning, although longer-term effects are unknown.^37,38^

### Strengths and limitations

This longitudinal study of a large representative sample of first-year undergraduate students attending a major Canadian university used validated measures to assess the importance of candidate distal and potentially modifiable proximal risk factors along with clinically significant symptoms at entry to university for mental health and academic outcomes at completion of the first year. Risk factors preceded outcomes and analyses adjusted for important confounders. Survey responses were linked to the university database to obtain an objective measure of academic performance. The overlap in mental health outcomes and their persistence from entry to completion of the academic year were examined, in addition to estimating rates. Further, multivariable regression models assessed the contribution of a variety of proximal risk factors to mental health outcomes, while adjusting for important distal risk factors along with all of the other predictors in the model. This study showed the high prevalence of persistent clinically significant mental health symptoms over the first year of university and identified specific risk factors associated with mental health and university-related outcomes that could be salient targets for universal prevention initiatives moving forward.

Strengths notwithstanding, several limitations should be mentioned. First, risk factors and mental health outcomes were measured by self-report, raising the possibility of recall error and information biases. Whereas anxiety and depressive symptoms are not diagnostic of a mental disorder, almost 50% of students who met screening thresholds also indicated a moderate to severe level of associated impairment, suggesting symptoms had clinical significance. The aim of this analysis was to estimate the importance of modifiable risk factors at entry to university to mental health and academic outcomes at the end of the first year, but did not address causative pathways or individualised risk prediction. In regard to the observed reduction in associations after adjustment, this was likely because of confounding with one or more of the other variables adjusted for in the model. Although it is outside the scope of this paper to investigate exactly which combinations of adjustments are most responsible for this change in the association, this would definitely be an interesting avenue for future research. Further, we limited the analysis to a finite set of candidate distal and potentially modifiable risk factors, and not all risk factors of importance were measured such as student debt; this may have led to unmeasured confounding. Also, given the attrition between entry and completion of the first year, selection bias may be present. Given the exploratory nature of this study, we did not adjust for multiple comparisons. Finally, these findings may not generalise to other university student populations and independent replication is planned.

### Implications

A substantial proportion of students entering university experience clinically significant anxiety and depressive symptoms and suicidal thoughts and behaviour that persist and are negatively associated with academic performance and university well-being. Given the ubiquitous nature of significant mental health symptoms in students, whole university approaches and investment in rigorous prevention and early intervention research, as set out in recent reports,^9,12,39^ seems justified. That is, universities should be mindful of potential system-level contributions to student stress related to the campus culture, scheduling of exams and graded assignments, and be encouraging of students to strike a healthy study–life balance; for example, by providing and ensuring access to subsidised art and cultural events, sporting and recreational programmes, and relaxation and mindfulness activities.

Although the provision of clinical care for students has not been the responsibility of universities, inadequacies in community mental health services to engage and serve a large diverse and transient help-seeking student population, often with illness severity below defined clinical thresholds, have compelled universities to take action. Moving forward universities, in collaboration with other responsible agencies and stakeholders, should develop a comprehensive student mental health strategy informed by the evidence. This strategy should encompass the provision of mental health literacy, prevention initiatives, timely assessment and when indicated care of students presenting with mild-to-moderate mental health conditions. Moreover, given the concentrated study terms and high-risk period for onset of severe and persistent mental disorders, university and community-based clinical programmes should develop a plan of facilitated transitions for students with moderate-to-severe mental illness.^7^

## Data Availability

Access to de-identified data from this study considered upon request to the corresponding author.

## Supplementary material

For supplementary material accompanying this paper visit http://dx.doi.org/10.1192/bjo.2020.24. click here to view supplementary material
