# Predictors of mental health and academic outcomes in first-year university students: Identifying prevention and early-intervention targets – CORRIGENDUM

**DOI:** 10.1192/bjo.2025.11

**Published:** 2025-02-03

**Authors:** A. Duffy, C. Keown-Stoneman, S. Goodday, J. Horrocks, M. Lowe, N. King, W. Pickett, S. H. McNevin, S. Cunningham, D. Rivera, L. Bisdounis, C. R. Bowie, K. Harkness, K. E. A. Saunders

In the original published version of this article, figure 1 was incorrect. The correct figure 1 is shown below.

**Fig. 1:**
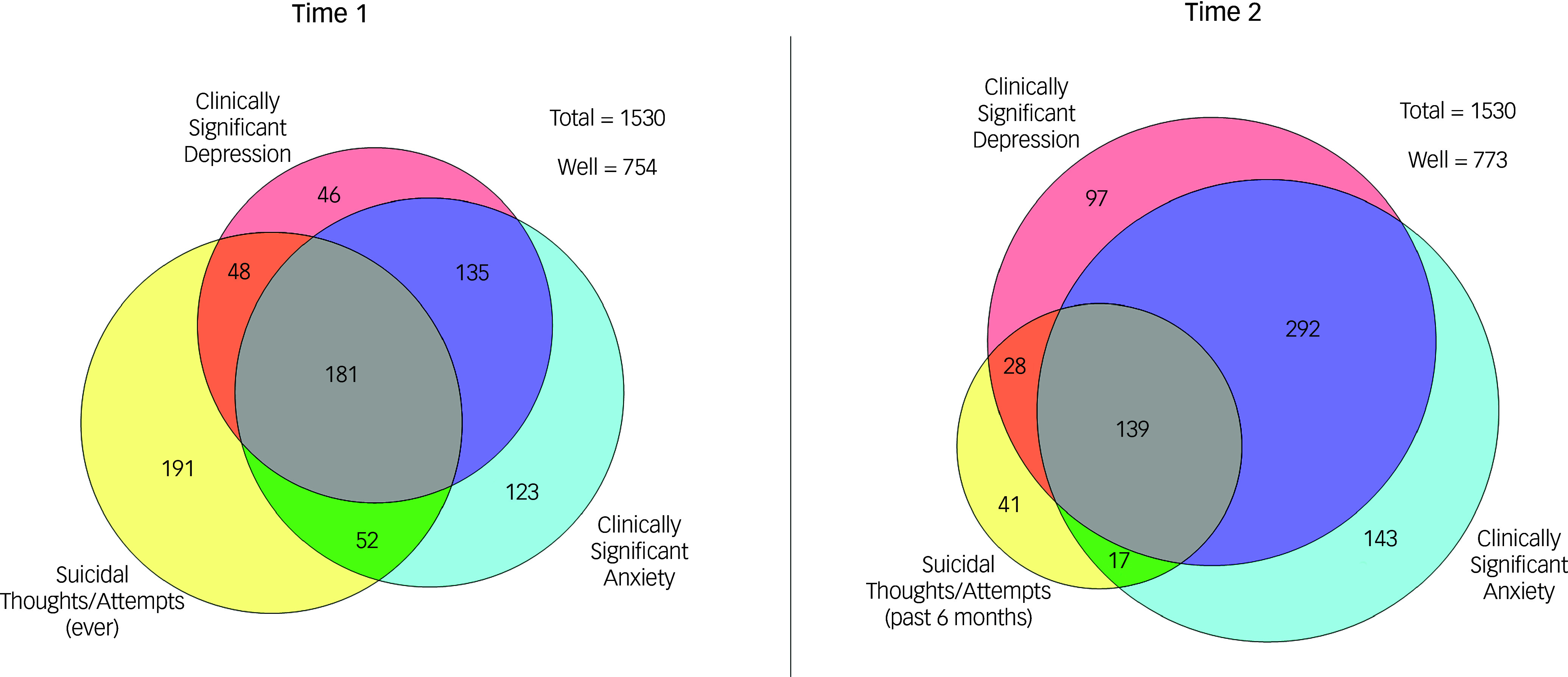
Overlap in mental health outcomes at Time 1 (entry to university) and Time 2 (end of first year).
