# Supplementary material for: Predictors of mental health and academic outcomes in first-year university students: Identifying prevention and early-intervention targets
Source: BJPsych Open. 2020 May 8;6(3):e46. doi: 10.1192/bjo.2020.24 (PMC7331085; doi:10.1192/bjo.2020.24)
Supplement: Supplementary file 1 [file S2056472420000241sup001.zip › S2056472420000241sup001/UFlourish MS Supplementary File BJP Feb 27.pdf]

Supplementary Tables and Figures

Supp. Table 1. Demographics of the baseline at entry to university (Time 1) and final analysis sample (complete Time 1 and Time 2 data)

	Time 1 sample (n= 3029)		Full analysis sample (n= 1530)		
	n	(%)[1]	n	(%)[1]	p-value[++]
Age					
≤17	586	(19.4)	319	(20.8)	0.10
18-19	2252	(74.4)	1136	(74.2)	
≥20	191	(6.3)	75	(4.9)	
Gender					
Male	993	(32.8)	400	(26.3)	<0.001
Female	2032	(67.2)	1120	(73.7)	
Ethnicity					
White	2000	(67.0)	1020	(66.7)	0.03
Asian	607	(20.3)	286	(18.7)	
Black	43	(1.4)	22	(1.4)	
Indigenous	9	(0.3)	0	(0.0)	
Other	43	(1.4)	19	(1.2)	
Multiple	285	(9.5)	183	(12.0)	
Parental Education Level[+]					
Degree in Professional School or Doctorate	691	(22.4)	185	(12.1)	<0.001
Master's degree	685	(23.8)	267	(17.5)	
Bachelor's degree or trades/apprenticeship	1131	(39.3)	748	(47.9)	
Completed high school or less	372	(12.9)	300	(19.6)	
Family history of mental disorder	1213	(40.0)	668	(43.7)	0.02
Lifetime history of mental disorder	779	(25.7)	404	(26.4)	0.64
Abused by a family member as child	201	(6.6)	100	(6.5)	0.95
Experienced sexual abuse as a child					
No	2262	(82.9)	1273	(83.2)	0.31
Yes	277	(10.1)	151	(9.9)	
Unsure	191	(7.0)	106	(6.9)	
Bullied by peers as a child	599	(19.8)	322	(21.8)	0.33

[1]percentage based on non-missing responses
[+] highest level for either parent; 45 participants did not give information about their parents' education
[++] p-value for chi-square test or Fisher's Exact test of equality of proportions between Time 1 and final analysis sample

Supplementary Tables and Figures

Supp. Table 2. Psychosocial and lifestyle predictors measured in the analysis sample at entry to university (Time 1) stratified by gender

Predictor (Time 1)	All (n=1530)		Male (n=400)		Female (n=1120)		M vs. F p-value+
Sleep Condition Indicator[a] *Mean (SD)*	22.23	(6.63)	23.92	(5.91)	21.69	(6.75)	<0.001
Clinically significant insomnia (SCI ≤ 16)	315	(20.6%)	51	(12.8%)	258	(23.0%)	<0.001
Hazardous substance use (more than once a week)[b]							
Any	727	(47.5%)	218	(54.5%)	505	(45.1%)	<0.01
Binge drinking	680	(44.5%)	208	(52.0%)	469	(41.9%)	<0.001
Use cannabis	140	(9.2%)	58	(14.6%)	81	(7.3%)	<0.001
Other substances	86	(5.7%)	22	(5.6%)	63	(5.7%)	1.00
Exercise (How often do you attend a gym or fitness class?)							<0.001
Never	243	(15.9%)	57	(14.2%)	183	(16.3%)	
Less than weekly	342	(22.4%)	66	(16.5%)	271	(24.2%)	
Once a week	342	(22.4%)	69	(17.2%)	271	(24.2%)	
2-3 times a week	401	(26.2%)	128	(32.0%)	273	(24.4%)	
4+ a week	202	(13.2%)	80	(20.0%)	122	(10.9%)	
Perceived stress total score[b] (maximum 16), *Mean (SD)*	6.76	(2.69)	5.90	(2.52)	7.06	(2.68)	<0.001
Self-esteem,[c] *Mean (SD)*	19.03	(5.38)	20.80	(5.17)	18.44	(5.30)	<0.001
<15 (low)	273	(17.8%)	39	(9.8%)	227	(20.3%)	<0.001
15-25 (normal)	1052	(68.8%)	283	(70.8%)	766	(68.4%)	
25+ (high)	205	(13.4%)	78	(19.5%)	127	(11.3%)	
Social support subscale score[b] (maximum 25), *Mean (SD)*	16.41	(3.82)	16.04	(3.96)	16.57	(3.76)	0.02

[+] *p-value for chi-square test or Fisher's Exact test of equality of proportions between males and females*
[a]*continuous scores on validated scales in including Sleep Condition Indicator, Brief Perceived Stress Scale, Rosenberg Self Esteem Scale, Social Support Subscale of the Adolescent Resiliency Scale (READ).* [b]*Engaging in at least one of the following once per week or more in the past month: binge drinking, use of non-prescribed sleep or wake-up pills, cannabis use, use of pain killers or opiates, and illicit drug use*

Supp Table 3. Unadjusted association between psychosocial and lifestyle predictors (Time 1) and depressive and anxiety symptoms (Time 1)++

	Depressive Symptoms (PHQ-9) Total Score (Time 1)				Anxiety Symptoms (GAD-7) Total Score (Time 1)			
**Predictor (Time 1)**	**beta**	**(95% CI)**	**p-value+**	**R^2**	**beta**	**(95% CI)**	**p-value+**	**R^2**
Self-esteem[a]	-0.13	(-0.14, -0.12)	<0.001	0.36	-0.12	(-0.13, -0.11)	<0.001	0.33
Substance use	0.10	(-0.02, 0.22)	0.09	0.002	0.01	(-0.11, 0.12)	0.91	<0.01
Exercise frequency[a]	-0.15	(-0.20, -0.11)	<0.001	0.03	-0.11	(-0.15, -0.06)	<0.001	0.01
Sleep quality[a]	-0.11	(-0.11, -0.10)	<0.001	0.37	-0.09	(-0.09, -0.08)	<0.001	0.24
Perceived stress[a]	0.27	(0.25 , 0.29)	<0.001	0.38	0.27	(0.26 , 0.29)	<0.001	0.40
Social support[a]	-0.08	(-0.09, -0.06)	<0.001	0.06	-0.06	(-0.07, -0.04)	<0.001	0.04

+p-value for test of association between outcome and predictor from univariate linear regression
[a]continuous predictor
++Lower self-esteem, less frequent exercise, poorer sleep quality, higher perceived stress and lower social support were associated with higher levels of self-reported anxiety and depressive symptoms at Time 1. For example, perceived stress explained approximately 40% of the variation in depressive (R^2=0.38) and anxiety (R^2=0.40) symptom scores, while sleep quality (R^2=0.37, R^2=0.24) and self-esteem (R^2=0.36, R^2=0.33) also explained substantial variation in depressive and anxiety symptom scores, respectively. Substance misuse at entry to university was not statistically associated with depressive or anxiety scores at Time 1.

Supp. Table 4. New onset vs. persistent binary clinical outcomes between Time 1 (at entry to university) and Time 2 (at completion of first year) within the analysis sample (n=1530)

	Time 1		Time 2 Total		Time 2 Persistent+		Time 2 New Onset++	
**Outcome**	**n**	**(%)**	**n**	**(%)**	**n**	**(% of Time 2 Total)**	**n**	**(% of Time 2 Total)**
Positive depression screen (PHQ-9 ≥10)	410	(26.8)	556	(36.3)	284	(51.1)	272	(48.9)
Positive anxiety screen (GAD-7 ≥10)	491	(32.1)	591	(38.6)	331	(56.0)	260	(44.0)
Suicidal ideation or attempts (yes)	472	(30.8)	225	(14.7)	178	(79.1)	47	(20.9)

+Persistent outcomes were defined as meeting screening thresholds at both entry (Time 1) and at the end of first year at university (Time 2);
++ New Onset outcomes were defined as meeting screening thresholds only at Time 2.

Supp. Table 5. Unadjusted and adjusted associations between distal factors and binary clinical outcomes measured at Time 1

	Positive Depression Screen (PHQ-9 ≥10) (n = 2489)					
	Unadjusted			Adjusted[a]		
**Predictor (pre-Time 1)**	**OR**	**(95% CI)**	**p-value+**	**OR**	**(95% CI)**	**p-value+**
Family history for mental illness	1.61	(1.36, 1.91)	<0.001	1.02	(0.83, 1.25)	0.86
Personal history of mental illness	3.12	(2.59, 3.76)	<0.001	2.38	(1.92, 2.93)	<0.001
Physically Abused	2.66	(1.98, 3.56)	<0.001	2.16	(1.55, 3.01)	<0.001
Bullied	2.65	(2.19, 3.21)	<0.001	1.92	(1.54, 2.39)	<0.001
Sexual Abuse: Yes	3.37	(2.61, 4.36)	<0.001	2.11	(1.58, 2.83)	<0.001
Sexual Assault: Unsure	2.80	(2.07, 3.79)	<0.001	1.99	(1.42, 2.77)	<0.001
	**Positive Anxiety Screen (GAD-7 ≥10) (n = 2490)**					
	**Unadjusted**			**Adjusted[a]**		
	**OR**	**(95% CI)**	**p-value+**	**OR**	**(95% CI)**	**p-value+**
Family history for mental illness	1.92	(1.63, 2.27)	<0.001	1.28	(1.06, 1.54)	0.01
Personal history of mental illness	3.41	(2.84, 4.09)	<0.001	2.57	(2.10, 3.15)	<0.001
Physically Abused	1.76	(1.31, 2.36)	<0.001	1.54	(1.10, 2.13)	0.01
Bullied	2.11	(1.75, 2.54)	<0.001	1.59	(1.28, 1.97)	<0.001
Sexual Abuse: Yes	2.21	(1.72, 2.85)	<0.001	1.23	(0.92, 1.65)	0.16
Sexual Abuse: Unsure	2.10	(1.55, 2.84)	<0.001	1.39	(1.00, 1.94)	0.05
	**Suicidal Thoughts or Attempts (yes) (n = 2490)**					
	**Unadjusted**			**Adjusted[a]**		
	**OR**	**(95% CI)**	**p-value+**	**OR**	**(95% CI)**	**p-value+**
Family history for mental illness	1.98	(1.67, 2.34)	<0.001	1.37	(1.12, 1.66)	<0.01
Personal history of mental illness	3.12	(2.60, 3.75)	<0.001	2.13	(1.73, 2.63)	<0.001
Physically Abused	3.34	(2.49, 4.50)	<0.001	2.66	(1.91, 3.72)	<0.001
Bullied	3.00	(2.48, 3.62)	<0.001	1.95	(1.57, 2.42)	<0.001
Sexual Abuse: Yes	4.19	(3.24, 5.43)	<0.001	2.91	(2.17, 3.90)	<0.001
Sexual Abuse: Unsure	2.71	(2.00, 3.66)	<0.001	1.82	(1.30, 2.53)	<0.001

+p-value for test of association between outcome and predictor from logistic regression
[a]Adjusted for: age, sex, and all other predictors listed in the table

Supp. Table 6a. Unadjusted and adjusted associations between psychosocial and lifestyle factors (Time 1) and *new onset* binary clinical outcomes (Time 2) using established clinical cut-offs (cut-off not already met at Time 1)

	New onset positive depression screen (PHQ-9 ≥10) (n = 1120)					
	Unadjusted			Adjusted[a]		
**Predictor (Time 1)**	**OR**	**(95% CI)**	**p-value+**	**OR**	**(95% CI)**	**p-value+**
Self esteem[b]	0.87	(0.84, 0.90)	<0.001	0.93	(0.89, 0.97)	<0.001
Substance use	1.26	(0.96, 1.65)	0.10	1.29	(0.95, 1.76)	0.11
Exercise[b]	0.95	(0.85, 1.05)	0.31	1.04	(0.93, 1.18)	0.48
Sleep[b]	0.90	(0.88, 0.93)	<0.001	0.94	(0.91, 0.97)	<0.001
Stress[b]	1.31	(1.23, 1.40)	<0.001	1.15	(1.07, 1.25)	<0.001
Social support[b]	0.96	(0.93, 1.00)	0.03	1.01	(0.97, 1.06)	0.65
	**New onset positive anxiety screen (GAD-7 ≥10) (n = 1039)**					
	**Unadjusted**			**Adjusted[a]**		
	**OR**	**(95% CI)**	**p-value+**	**OR**	**(95% CI)**	**p-value+**
Self esteem[b]	0.90	(0.87, 0.92)	<0.001	0.96	(0.92, 1.00)	0.06
Substance use	1.18	(0.89, 1.56)	0.26	0.99	(0.72, 1.35)	0.94
Exercise[b]	1.00	(0.90, 1.12)	0.97	1.14	(1.01, 1.30)	0.04
Sleep[b]	0.90	(0.88, 0.93)	<0.001	0.94	(0.91, 0.96)	<0.001
Stress[b]	1.32	(1.23, 1.41)	<0.001	1.20	(1.11, 1.31)	<0.001
Social support[b]	0.97	(0.94, 1.01)	0.11	1.02	(0.98, 1.07)	0.28
	**New onset suicidal thoughts or attempts (yes) (n = 1058)**					
	**Unadjusted**			**Adjusted[a]**		
	**OR**	**(95% CI)**	**p-value+**	**OR**	**(95% CI)**	**p-value+**
Self esteem[b]	0.89	(0.83, 0.94)	<0.001	0.93	(0.86, 1.01)	0.09
Substance use	1.37	(0.76, 2.47)	0.30	1.43	(0.76, 2.72)	0.27
Exercise[b]	0.85	(0.67, 1.07)	0.16	0.92	(0.71, 1.18)	0.49
Sleep[b]	0.93	(0.89, 0.97)	<0.01	0.97	(0.92, 1.02)	0.23
Stress[b]	1.22	(1.09, 1.37)	<0.001	1.06	(0.91, 1.24)	0.46
Social support[b]	0.95	(0.89, 1.01)	0.08	0.98	(0.91, 1.06)	0.54

+p-value for test of association between outcome and predictor from logistic regression
[a]Adjusted for: age, sex, physical and sexual abuse, bullied, family history for mental illness, personal history of mental illness and all other predictors listed in the table
[b]continuous predictor

Supp. Table 6b. Unadjusted and adjusted associations between psychosocial and lifestyle factors (Time 1) and *persistent* binary clinical outcomes using established clinical cut-offs (cut-off met at both Time 1 and Time 2)

	**Persistent positive depression screen (PHQ-9 ≥10) (n = 410)**					
	**Unadjusted**			**Adjusted[a]**		
**Predictor (Time 1)**	**OR**	**(95% CI)**	**p-value+**	**OR**	**(95% CI)**	**p-value+**
Self esteem[b]	0.88	(0.83, 0.92)	<0.001	0.88	(0.82, 0.93)	<0.001
Substance use	1.04	(0.68, 1.58)	0.86	1.19	(0.75, 1.92)	0.46
Exercise[b]	0.90	(0.76, 1.07)	0.23	0.89	(0.73, 1.08)	0.22
Sleep[b]	0.94	(0.91, 0.97)	<0.001	0.95	(0.92, 0.99)	0.01
Stress[b]	1.11	(1.01, 1.22)	0.03	0.92	(0.82, 1.04)	0.17
Social support[b]	0.94	(0.89, 0.99)	0.03	0.97	(0.91, 1.03)	0.37
	**Persistent positive anxiety screen (GAD-7 ≥10) (n = 491)**					
	**Unadjusted**			**Adjusted[a]**		
	**OR**	**(95% CI)**	**p-value+**	**OR**	**(95% CI)**	**p-value+**
Self esteem[b]	0.90	(0.86, 0.94)	<0.001	0.94	(0.89, 1.00)	0.05
Substance use	0.83	(0.57, 1.21)	0.32	0.92	(0.61, 1.41)	0.71
Exercise[b]	0.83	(0.71, 0.96)	0.01	0.85	(0.72, 1.00)	0.05
Sleep[b]	0.94	(0.91, 0.97)	<0.001	0.96	(0.92, 0.99)	0.01
Stress[b]	1.21	(1.11, 1.32)	<0.001	1.07	(0.96, 1.20)	0.22
Social support[b]	0.94	(0.89, 0.99)	0.03	0.97	(0.92, 1.03)	0.39
	**Persistent suicidal thoughts or attempts (Yes) (n = 472)**					
	**Unadjusted**			**Adjusted[a]**		
	**OR**	**(95% CI)**	**p-value+**	**OR**	**(95% CI)**	**p-value+**
Self esteem[b]	0.84	(0.80, 0.88)	<0.001	0.86	(0.81, 0.91)	<0.001
Substance use	1.05	(0.72, 1.53)	0.80	1.03	(0.67, 1.58)	0.90
Exercise[b]	0.81	(0.70, 0.93)	<0.01	0.89	(0.75, 1.05)	0.18
Sleep[b]	0.96	(0.93, 0.98)	<0.001	1.00	(0.97, 1.03)	1.00
Stress[b]	1.29	(1.19, 1.40)	<0.001	1.09	(0.98, 1.21)	0.10
Social support[b]	0.95	(0.91, 1.00)	0.05	1.02	(0.97, 1.09)	0.45

+p-value for test of association between outcome and predictor from logistic regression
[a]Adjusted for: age, sex, physical and sexual abuse, bullied, family history for mental illness, personal history of mental illness and all other predictors listed in the table
[b]continuous predictor

Supp. Table 7. Unadjusted and adjusted associations between psychosocial and lifestyle predictors (Time 1) and continuous clinical outcomes at (Time 2)

	Depressive Symptoms[c] (PHQ-9 total score)					
	Unadjusted			Adjusted[a]		
**Predictor (Time1)**	**beta**	**(95% CI)**	**p-value***	**beta**	**(95% CI)**	**p-value***
Self esteem[b]	-0.11	(-0.12, -0.10)	<0.001	-0.05	(-0.06, -0.03)	<0.001
Substance use	0.15	(0.02, 0.27)	0.02	0.13	(0.02, 0.23)	0.02
Exercise[b]	-0.12	(-0.17, -0.07)	<0.001	-0.02	(-0.07, 0.02)	0.26
Sleep quality[b]	-0.09	(-0.09, -0.08)	<0.001	-0.05	(-0.06, -0.04)	<0.001
Stress[b]	0.21	(0.19, 0.23)	<0.001	0.07	(0.04, 0.10)	<0.001
Social support[b]	-0.07	(-0.08, -0.05)	<0.001	-0.01	(-0.03, 0.002)	0.10
	**Anxiety Symptoms[c] (GAD-7 total score)**					
	**Unadjusted**			**Adjusted[a]**		
	**beta**	**(95% CI)**	**p-value***	**beta**	**(95% CI)**	**p-value***
Self esteem[b]	-0.10	(-0.11, -0.09)	<0.001	-0.03	(-0.05, -0.02)	<0.001
Substance use	0.16	(0.04, 0.29)	0.01	0.14	(0.03, 0.24)	0.01
Exercise[b]	-0.08	(-0.13, -0.04)	<0.001	0.01	(-0.03, 0.05)	0.58
Sleep quality[b]	-0.08	(-0.09, -0.07)	<0.001	-0.04	(-0.05, -0.03)	<0.001
Stress[b]	0.22	(0.20, 0.24)	<0.001	0.11	(0.09, 0.14)	<0.001
Social support[b]	-0.05	(-0.06, -0.03)	<0.001	-0.0003	(-0.015, 0.014)	0.97

**p-value test of association between outcome and predictor*
GAD-7: Generalized Anxiety Disorder Scale; PHQ-9: Patient Health Questionnaire
[a]Adjusted for: age, gender, physical and sexual abuse, bullied, family history for mental illness, personal history of mental illness and all other predictors listed in the table
[b]Continuous variable
[c]Outcome was normalized with a square root transformation

Supplementary Tables and Figures

Supp. Figure 1. Sample Selection

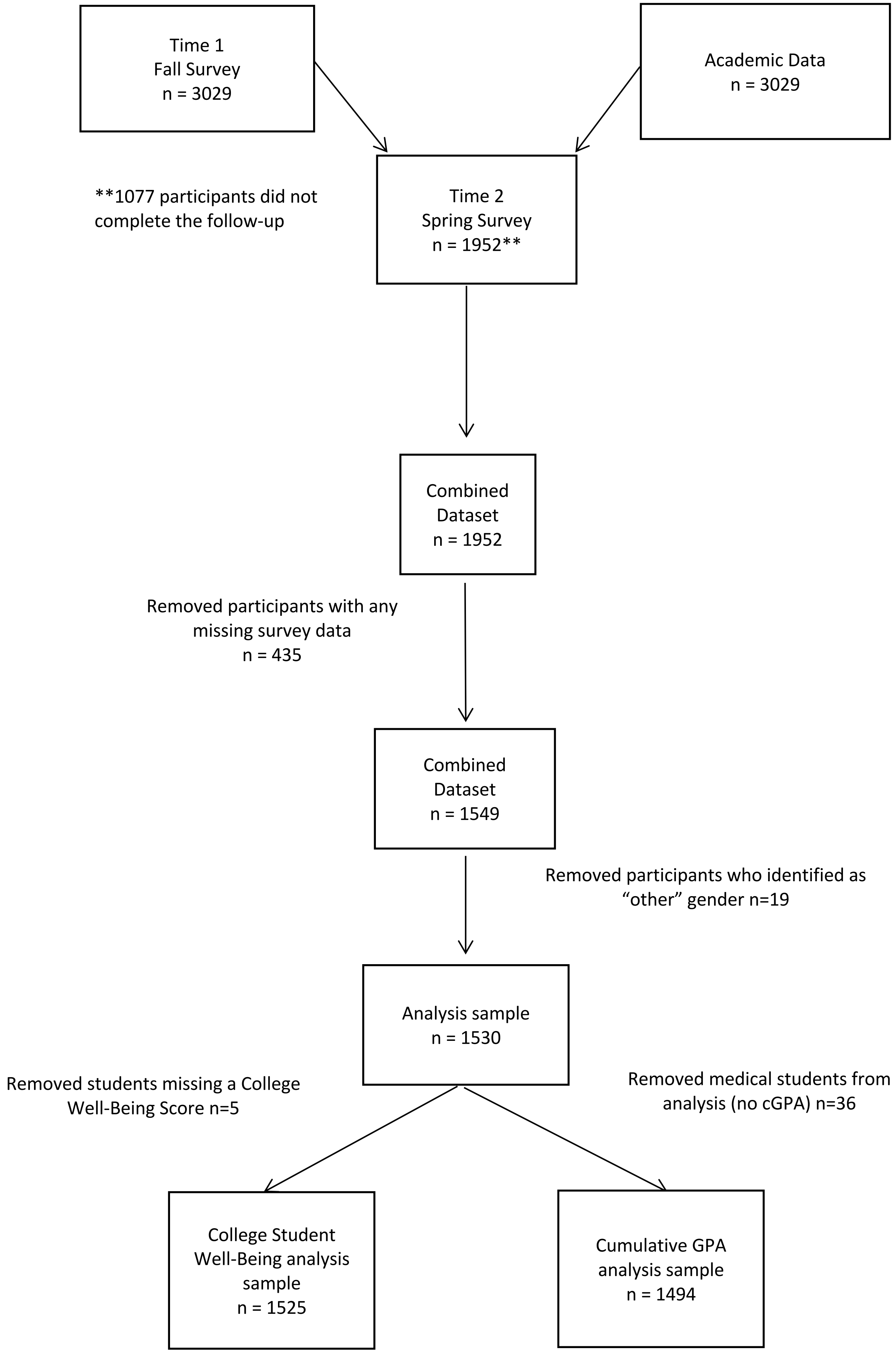

Supp. Figure 2. Depressive and anxiety symptoms at university entry (Time 1) and completion of first year (Time 2)

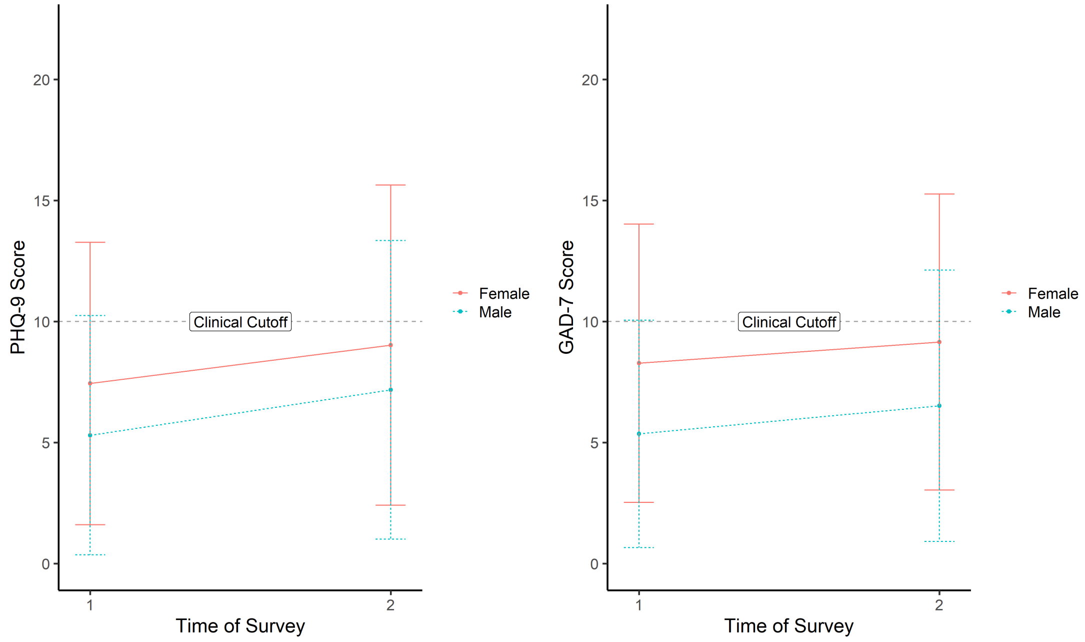

Patient health questionnaire (PHQ-9) measuring depressive symptoms; generalized anxiety disorder scale (GAD-7) measuring anxiety; males (blue) and females (red); error bars show 1 standard deviation above and below the mean

Supp. Figure 3. Proportion of students screening positive for mental health outcomes at Time 1 and Time 2

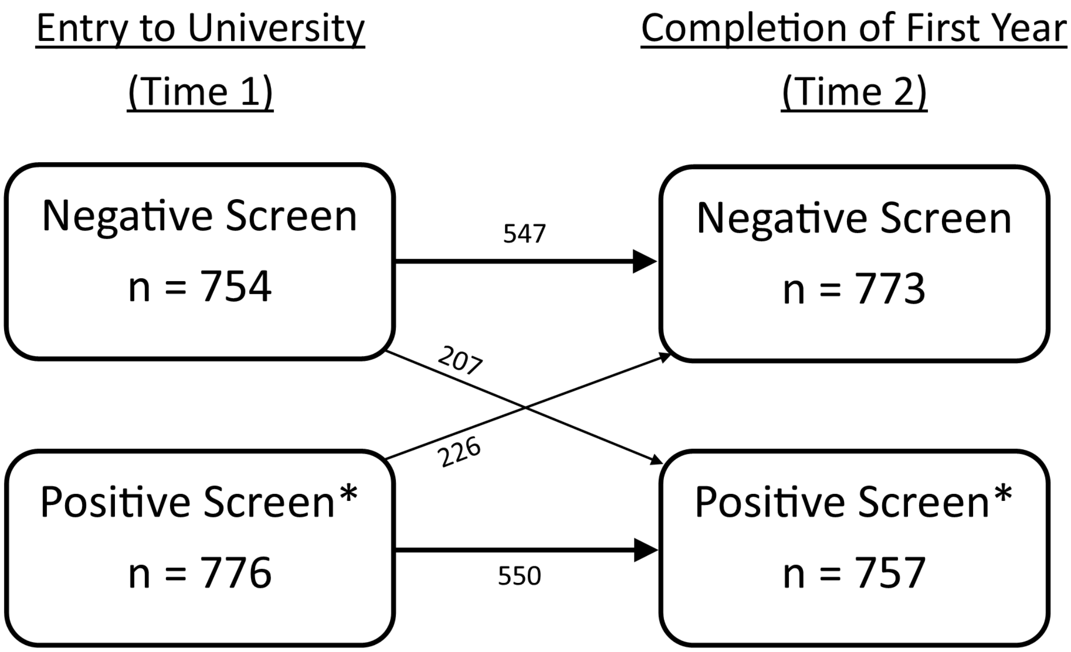

**Clinically significant anxiety, and/or depressive symptoms, and/or suicidal thoughts or attempts*
